# Reply to the comments of Naharci *et al*. on “Circulating level of fatty acid‐binding protein 4 is an independent predictor of metabolic dysfunction‐associated fatty liver disease in middle‐aged and elderly individuals”

**DOI:** 10.1111/jdi.13808

**Published:** 2022-05-07

**Authors:** Marenao Tanaka, Masato Furuhashi

**Affiliations:** ^1^ Department of Cardiovascular, Renal and Metabolic Medicine Sapporo Medical University School of Medicine Sapporo Japan

We appreciate Naharci *et al.* ^1^ for their interest and constructive comments on our recent article^2^, which investigated associations between metabolic dysfunction‐associated fatty liver disease (MAFLD) and fatty acid‐binding protein 4 (FABP4) in a Japanese general population. In their recent letter to the editor, the authors claimed that physical activity level and renal function were not taken into consideration as potential confounders^1^ .

FABP4 is mainly expressed in both adipocytes and macrophages and acts as an adipokine for the development of insulin resistance and atherosclerosis, resulting in a possible therapeutic target for metabolic and cardiovascular diseases^3^. It has been reported that an elevated circulating FABP4 level is associated with several metabolic disorders including obesity^3^. It has also been shown that the FABP4 concentration can be a predictor for the development of type 2 diabetes mellitus, atherosclerosis, and cardiovascular events^3^. Since FABP4 is highly regulated by adiposity^3^, physical activity may affect the FABP4 concentration as well as the severity of hepatosteatosis in MAFLD. However, unfortunately, we could not include physical activity as a confounder in multivariable logistic regression analyses for MAFLD since detailed information about physical activity was not obtained in our recent study^2^ .

We previously showed that FABP4 expression was ectopically induced in glomerular endothelial cells and macrophages by glomerular injury and that the extent of glomerular FABP4 expression was associated with renal dysfunction and proteinuria^4^. Furthermore, we showed that the urinary FABP4 (U‐FABP4) level was associated with albuminuria and predicted a decline of estimated glomerular filtration rate (eGFR) during a 1 year follow‐up period in a population‐based study^5^ and a cohort study using nephrotic patients who underwent kidney biopsy^6^, suggesting that the excretion of U‐FABP4 can be a novel biomarker of glomerular damage. It has also been reported that an elevated circulating FABP4 level is strongly associated with renal dysfunction, probably due to the reduced renal elimination of FABP4^3^. Furthermore, a possible link between chronic kidney disease and MAFLD has been suggested. Therefore, it is possible that renal dysfunction affects both FABP4 concentration and MAFLD.

In logistic regression analyses, the FABP4 concentration was independently associated with MAFLD after adjustment of age, sex, presence of hypertension, diabetes mellitus, and dyslipidemia, and the levels of adiponectin, fibroblast growth factor 21, uric acid and HOMA‐R (Table 5, Model 4 in our recent study^2^) and after adjustment of eGFR in addition to Model 4 (Table 1 in this letter). In conclusion, the FABP4 concentration is an independent predictor of MAFLD in middle‐aged and elderly individuals after adjustment of several confounders including renal function.

**Table 1:** | Multivariable logistic regression analysis for the risk of MAFLD

	OR (95% CI)	*P*
FABP4 (per 1 μg/L)	1.066 (1.020–1.091)	<0.001
Adiponectin (per 1 mg/L)	0.954 (0.916–0.993)	0.013
FGF21 (per 1 ng/L)	1.001 (1.000–1.003)	0.022
Age (per 1 year)	0.998 (0.980–1.015)	0.778
Sex (Men)	0.435 (0.275–0.689)	<0.001
Hypertension	2.115 (1.362–3.284)	0.001
Diabetes mellitus	1.075 (0.576–2.004)	0.821
Dyslipidemia	1.928 (1.317–2.823)	0.001
Uric acid (per 1 mg/dL^†^)	1.378 (1.150–1.649)	0.001
HOMA‐R (per 1)	1.082 (1.020–1.147)	0.009
eGFR ((per 1 mL/min/1.73m^2^)	1.024 (1.006–1.043)	0.009
	*R* ^2^ = 0.176, AIC: 696	

^†^ 59.48 μmol/L. AIC, Akaike's information criterion; CI, confidence Interval; eGFR, estimated glomerular filtration rate; FABP4, fatty acid‐binding protein 4; FGF21, fibroblast growth factor 21; HOMA‐R, homeostasis model assessment of insulin resistance; MAFLD, metabolic dysfunction‐associated fatty liver disease; OR, odds ratio.

## DISCLOSURE

The authors declare no conflict of interest.

Approval of the research protocol: N/A.

Informed consent: N/A.

Registry and the registration no. of the study/trial: N/A.

Animal studies: N/A.
